# Posterior reversible encephalopathy syndrome in an adult patient undergoing peritoneal dialysis: a case report and literature review

**DOI:** 10.1186/1471-2369-15-10

**Published:** 2014-01-13

**Authors:** Brett R Graham, George B Pylypchuk

**Affiliations:** 1 Department of Medicine, Division of Neurology, University of Saskatchewan, Room 3544 RUH, 103 Hospital Drive, Saskatoon, SK, Canada; 2 Department of Medicine, Division of Nephrology, University of Saskatchewan, Room 3544 RUH, 103 Hospital Drive, Saskatoon, SK, Canada

**Keywords:** Peritoneal dialysis, Posterior reversible encephalopathy syndrome, End-stage renal disease, Hypertension

## Abstract

**Background:**

Posterior reversible encephalopathy syndrome (PRES) is a clinical and radiological entity characterized clinically by headache, altered mental status, seizures, visual disturbances, and other focal neurological signs, and radiographically by reversible changes on imaging. A variety of different etiologies have been reported, but the underlying mechanism is thought to be failed cerebral autoregulation. To the best of our knowledge, we report the third known case of PRES in an adult receiving intermittent peritoneal dialysis (PD).

**Case presentation:**

A 23-year-old male receiving PD was brought to hospital after experiencing a generalized seizure. On presentation he was confused and hypertensive. An MRI brain was obtained and showed multiple regions of cortical and subcortical increased T2 signal, predominantly involving the posterior and paramedian parietal and occipital lobes with relative symmetry, reported as being consistent with PRES. A repeat MRI brain obtained three months later showed resolution of the previous findings.

**Conclusion:**

Due to having a large number of endothelium-disrupting risk factors, including hypertension, uremia, and medications known to disrupt the cerebrovascular endothelium, we suggest that those with end-stage renal disease (ESRD) receiving PD are at high risk of developing PRES. Furthermore, we surmise that PRES is likely more prevalent in the ESRD population but is under recognized. Physicians treating those with ESRD must have a high index of suspicion of PRES in patients presenting with neurological disturbances to assure timely diagnosis and treatment.

## Background

Posterior reversible encephalopathy syndrome (PRES) is a clinical and radiological condition that involves a variety neurological manifestations and reversible white matter changes on T2-weighed MRI sequences. PRES has its origins in another acronym, RPLS (reversible posterior leukoencephalopathy syndrome), coined by Hinchey and colleagues in 1996 [1]. The term then evolved to emphasize the involvement of both grey and white matter, and to make the acronym more memorable (as it has been long thought that PRES is caused by high blood PRESsure) [2].

Case reports regarding PRES in patients receiving PD are scarce. We propose that patients with end-stage renal disease (ESRD) receiving PD are at a higher risk of developing PRES due to both cerebral dysregulation and endothelial dysfunction, secondary to complications of ESRD and side-effects of medications they receive. Furthermore, we surmise that PRES is likely more prevalent than previously thought in the ESRD population, and only extreme cases heralded by seizure are brought to medical attention, or alternatively, recognized by healthcare practitioners.

## Case presentation

A 23-year old male with ESRD secondary to IgA nephropathy was brought to home hospital for generalized seizure activity. He was known to have poor compliance with his intermittent peritoneal dialysis (PD), administer four times per day, and had apparently missed his last two treatments. He did not have a recent peritoneal equilibrium test. On arrival to the hospital he was found to have a blood pressure exceeding 182/107 mmHg and was described as being combative and confused. Labetalol 20 mg IV, lorazepam 1 mg IV, and haloperidol 1 mg IV were administered by a nurse practitioner before being transferred to the nearest tertiary care centre. Collateral history obtained suggested that the patient was complaining of headache and blurred vision hours prior to seizing.

Other past medical history was significant for hypertension secondary to renovascular disease, as well as being a carrier of methicillin-resistant staphylococcus aureus. His medications included atenolol, amlodipine, ramipril, hydroxyzine, scheduled clonidine, erythropoetin, calcium, and Replivite tablets. There was no family history of epilepsy. Social history was significant for tobacco smoking and previous recreational cocaine use.

Examination at the tertiary centre revealed a temperature of 36.8°C and a still elevated blood pressure of 200/100 mmHg. Heart rate was recorded at 63 beats per minute and oxygen saturation was 98% on room air. General examination revealed a rousable individual who appeared somewhat confused, but was alert to person and place. The remaining neurological exam, including language assessment and fundoscopy, was normal. There was no meningismus. Cardiovascular exam was significant for a loud S2 heart sound, but no cardiac rubs or murmurs were appreciated. Respiratory and abdominal exams were non-contributory.

Admission blood work revealed a urea of 41 mmol/L, a serum creatinine of 1531 μmol/L, a WBC of 11.4, and hemoglobin of 99. Serum sodium was slightly low at 129 mol/L, and potassium was elevated at 6.3 mmol/L. Calcium was low at 1.93 mmol/L, phosphate was elevated at 3.57 mmol/L, and magnesium was 1.24 mmol/L. Liver enzymes were generally unremarkable. An EKG revealed tall peaked T-waves consistent with hyperkalemia. A CT-head showed multiple cortical and subcortical hypodensities involving the posterior parasagittal portions of both parietal lobes and the medial right occipital lobe, which was reported as being suggestive of PRES. A lumbar puncture for CSF analysis to rule out meningoencephalitis was not performed as the patient was afebrile with no signs of meningeal irritation.

**Figure 1:**
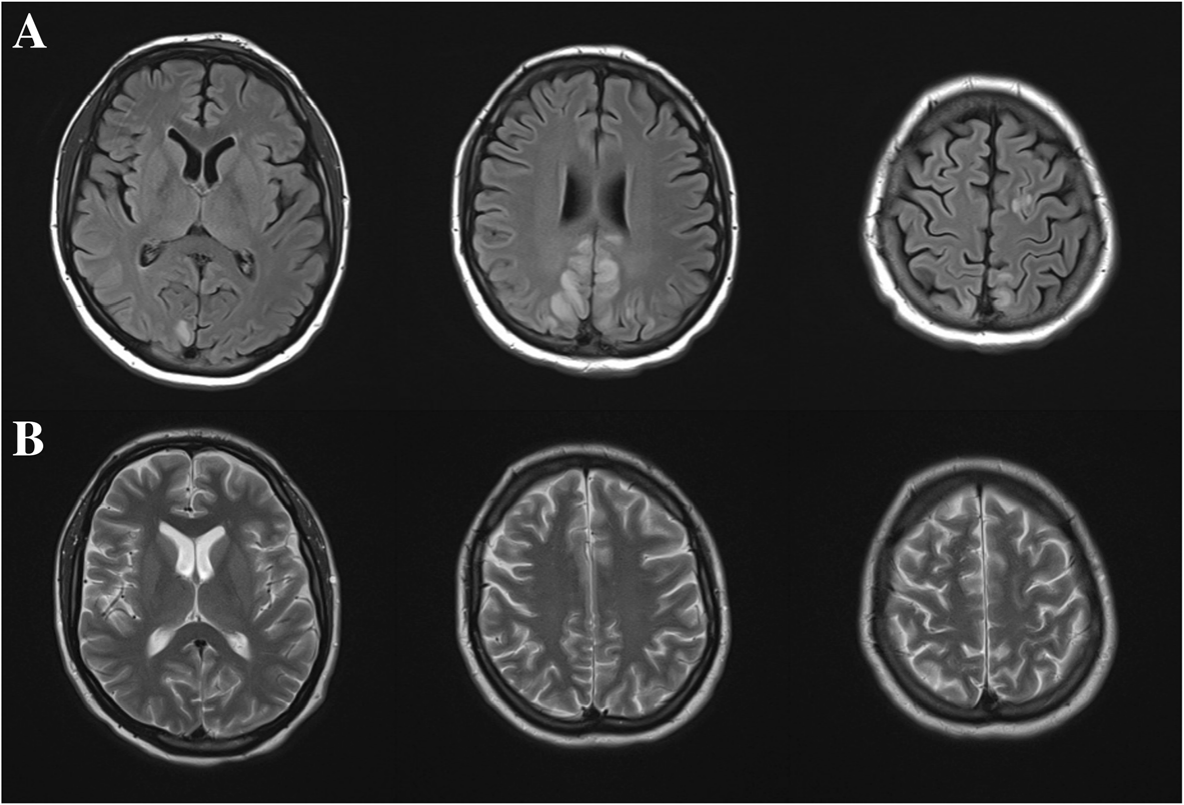
**Axial MRI brain of patient.** In panel **A**, there are T2-FLAIR hyperintensities involving the paramedian parietal and occipital lobes, suggesting vasogenic edema. T2-weighted images in panel **B** were taken three months after discharge and show a resolution of the abnormalities, confirming the diagnosis of PRES.

The patient was admitted to hospital for further work-up and management. The hyperkalemia was medically treated. Phenytoin was initiated to prevent further seizures. An MRI brain was obtained and showed multiple regions of cortical and subcortical increased T2 signal, predominantly involving the posterior and paramedian parietal and occipital lobes with relative symmetry (Figure 1A). There was no diffusion restriction. On the third day after admission the patient began to have episodes of headache and blurred vision associated with increased blood pressure, despite achieving good volume control through in-hospital PD and increasing his antihypertensive regimen. These episodes continued, and thus on the fifth day after admission he was transferred to the intensive care unit to receive continuous blood pressure monitoring and control through an intravenous labetalol infusion. Blood pressure was decreased to 160/80 mmHg and he was transitioned back to oral antihypertensive medication. He was subsequently discharged from hospital and follow-up MRI brain three months later showed complete resolution of previously noted findings (Figure 1B).

## Discussion

Clinically, PRES is characterized by visual disturbances, headache, nausea, change in mental status, and seizure. Seizures are cited as the commonest manifestation of PRES, occurring in up to 90% of reported cases [3,4]. Seizure type is mainly generalized, but focal onset has been reported [5]. Visual disturbances can range from complaints of blurred vision to cortical blindness [5]. Symptoms develop over hours and can persist for weeks, depending on the severity and the latency in initiating proper treatment.

Acute hypertension is also associated with the majority of PRES cases, but is not necessary for the diagnosis, and the degree of elevation varies [6]. Cases with normal or low-normal blood pressure values have been reported [7-9], but it has been suggested that the rapid increase in blood pressure rather than the absolute value delivers the most risk [6,10]. Bartynski and Boardman [11] found no correlation between absolute blood pressure and clinical and radiographic presentations of PRES.

Radiographically, PRES is heralded by relatively symmetric, reversible T2 hyperintensities affecting the posterior aspects of the brain, namely the occipital and parietal lobes [1]. It is now known that this description is more of a general rule, and that asymmetric pictures can be seen, and can involve the deep grey matter as well as the frontal and temporal lobes [12]. The advent of diffusion weighted imaging helped clarify that the MRI changes were not due to ischemia or cytotoxic edema, but due to vasogenic edema [13]. However, there are cases that involve some degree of diffusion restriction, suggesting that ischemia can be a complication of PRES [13].

Bartynski and Boardman [11] described four primary variations of the typical PRES imaging pattern after reviewing the imaging of 136 patients with PRES. Practically all patients (134/136) had the classic posterior occipital or parietal pattern, but differences were seen in the involvement of the frontal lobes and other structures. A holohemispheric watershed pattern was seen in 31 patients, where vasogenic edema followed the watershed territories between the main cerebral arteries. A superior frontal sulcus pattern was seen in 37 patients, where vasogenic edema stretched forward along the superior frontal sulcus, sparring the extreme frontal poles. A dominant parietal-occipital pattern was also described in 30 patients, where there was no involvement of the frontal lobes and variable involvement of the temporal lobes. Partial expressions of the primary patterns were also noted in 38 of the patients and atypical lesions involving the basal ganglia, brainstem, and deep white matter were seen in a minority of patients.

The incidence of PRES is unknown, but it has been reported worldwide with a slight female predominance [5,11,14]. PRES can affect both young and old, with the mean age falling within the third or fourth decade. It is traditionally thought as a monophasic illness, but recurrent cases have been reported [15]. Underlying comorbidities are usually present, contributing to acute hypertension or endothelial dysfunction, or both. A recent retrospective review of PRES cases in German hospitals found acute renal failure, immunosuppression, organ transplant, infection, and autoimmune disorder, among others, to be common associated comorbidities in PRES [14].

The differential diagnosis of PRES is broad, given its non-specific clinical manifestations, and it needs to be distinguished from other causes of headache, confusion, and seizure. Severe neurological conditions such as encephalitis, posterior circulation stroke, cerebral venous sinus thrombosis, reversible cerebral vasoconstriction syndrome, and ictal and post-ictal states can have similar clinical presentations as PRES and need to be considered as possible diagnoses. PRES should be considered in all patients presenting with an acute confusional state associated with elevated blood pressure and/or an underlying cause of endothelial dysfunction [5,6,16].

The putative pathophysiology behind PRES is that of failed cerebral autoregulation. Cerebral blood flow (CBF) is maintained at a constant level across a range of mean arterial pressures (MAP) by way cerebral autoregulation [17]. Cerebral autoregulation is achieved by vasoconstriction or dilation of resistance arterioles, resulting in relatively consistent blood flow to the brain. Vasoconstriction and dilation are thought to be caused by a direct myogenic response [18], and it is when the thresholds of adaptation are surpassed that cerebral dysregulation occurs. When resistance arterioles can no longer respond to increasing MAPs, CBF can increase unchecked, leading to elevation of filtration pressures in the capillary beds, and resulting in extrasavation of plasma in the surrounding brain parenchyma leading to cerebral edema [17]. The posterior circulation is more at risk of developing edema due to decreased sympathetic innervation as compared to the anterior circulation [19], and thus a decreased ability to autoregulate.

Cerebral dysregulation can also result in the breakdown of the blood-brain barrier (BBB). The BBB consists of vascular endothelium with tight intercellular junctions, a basement membrane, and surrounding glial cells. Animal studies have shown that the vascular endothelium is the main barrier in keeping fluids and molecules in their respective intra- and extravascular spaces [20,21]. The extravasation of plasma occurs through the tight junctions as well as through pinocytosis in experimentally-induces hypertension [21]. From this, the cerebral dysregulation seen in PRES can be a consequence of increased filtration pressure at the level of the capillary beds, which in turn overcomes the vascular endothelium and tight intercellular junctions of the BBB. The result is endothelial dysfunction and surrounding vasogenic edema.

Endothelial dysfunction may actually be an independent variable in the development of PRES. There are reports of PRES with normal or minimally elevated blood pressures [7-9], which do not fit within the paradigm of an overwhelmed cerebral autoregulation. These cases, however, involved a systemic illness (e.g. sepsis or eclampsia) or introduction of a medication (e.g. cyclosporine A) that is thought to disrupt the BBB. The disruption of the BBB allows seepage of fluid into the surrounding brain tissues, resulting in cerebral edema that is clinically and radiographically indistinct from PRES. It has also been postulated that damaged endothelium can impair cerebral autoregulation and thus lower the threshold for dysregulation [6]. It seems as if PRES can result from increases in blood pressure that overcomes cerebral autoregulation and the BBB, or it can occur independently in conditions that damage or impair the BBB.

Patients with ESRD likely have baseline endothelial dysfunction secondary to ongoing uremia [22]. Endothelial dysfunction may impede cerebral autoregulation, allowing for extravasation of fluid at much lower MAPs, resulting in vasogenic edema, and subsequently PRES. Indeed, PRES has been reported in cases of uremic encephalopathy with near normal blood pressures [23,24], again suggesting that endothelial dysfunction is an independent variable for developing PRES.

A number of medications have been implicated in inducing PRES, including erythropoietin [25], which is commonly prescribed to ESRD patients to treat the associated normocytic anemia. Erythropoietin works by stimulating the bone marrow to release more reticulocytes [26], and is known to induce or exacerbate hypertension in ESRD patients [27]. This worsening of blood pressure may play a role cerebral dysregulation, and ultimately PRES.

Medication withdrawal has also been implicated in the development of PRES. Nakabou and colleagues report a case of PRES following the discontinuation of antihypertensive medication in a man with ESRD [28], and resolution of symptoms when antihypertensives were restarted. Clonidine, which can cause a rebound hypertension if discontinued quickly [29], has also been implicated in PRES [6]. It is possible that our patient was not taking his medication in the hours leading up him presentation, resulting in a rebound hypertension, and thus exacerbating a process already underway.

There is a myriad of literature of PRES occurring in conditions that cause acute renal failure, but only four cases of PRES occurring in patients with ESRD receiving PD. Kitamura and colleagues [30] reported the first case of PRES in an adult patient receiving PD. The case involved a 24-year-old male who presented with decreased level of consciousness and generalized tonic-clonic seizure. Blood pressure was measured at 170/80 mmHg, and medications included erythropoietin. The authors surmised that the root cause of PRES in his case was poor compliance with his PD and blood pressure medications. Symptoms resolved with aggressive blood pressure and volume control. Abnormalities initially found on MRI brain had resolved when follow-up imaging was undertaken one-month later.

Ogawa and colleagues [31] describe a case of PRES in a 24-year-old Japanese woman receiving PD. She presented with severe headache, generalized seizures, and decreased level of consciousness. Systolic blood pressure was reported at being around 200 mmHg the morning of the incident. The patient’s clinical picture improved with aggressive antihypertensive therapy and increased PD dose. Initial MRI abnormalities normalized 8 days after admission.

Girisgen and colleagues [15] describe recurrent PRES in a young boy receiving PD. He presented with headache and seizure, and the authors were concerned about an underlying infection. Blood pressure was initially measured at 135/95 mmHg. The second instance of PRES was associated with a presenting blood pressure of 160/100 mmHg, but no sign of underlying infection. With blood pressure and volume control, discontinuation of erythropoietin, and treatment of a presumed underlying infection in the first presentation, the patient’s seizures and visual symptoms abated and follow-up imaging normalized one month after each instance of PRES. As the boy was on PD for over a decade, the occurrence of PRES could not be linked to the initiation of dialysis.

Delanty and colleagues [25] provide a case of PRES in a 12-year-old boy who had been receiving PD for approximately four months. He presented with headache, lethargy, visual disturbances, and generalized tonic-clonic seizure. Blood pressure on presentation was 220/120 mmHg and medications included erythropoietin. Blood pressure normalized and clinical symptoms abated when erythropoietin was withdrawn.

All four of these cases share disturbed level of consciousness and seizure as part of their presentation, which is consistent with the PRES literature [3,4]. All patients were hypertensive on presentation as well, save for the initial presentation of the young boy as described by Girisgen and colleagues [15], who had a blood pressure of 135/95 mmHg. The development of PRES in this case could have been due to underlying endothelial dysfunction at the time, since it was thought that the boy had an underlying infection. Not surprisingly, three of the four patients were taking erythropoietin, which may have contributed to their development of PRES. Interestingly, one of the cases described an improvement in blood pressure and resolution of symptoms simply by discontinuing erythropoietin [25]. Blood pressure control was paramount in all cases for the resolution of symptoms and the reversal of imaging abnormalities. Volume control was a significant factor in managing blood pressure in two of the cases [15,30] (Table 1).

**Table 1:** Clinical summary of PRES in PD patients

**Case study**	**Age and sex**	**Presentation**	**Blood pressure**	**Medications implicated in PRES**	**Presumed underlying cause**	**Treatment**
**Kitamura *et al *,**[30]	24 yo male	Decreased LOC, seizure	170/80 mmHg	Erythropoietin	Poor compliance with PD and medication	BP management and volume control (PD and HD)
**Ogawa *et al *,**[31]	24 yo female	HA, generalized seizure, decreased LOC	190/117 mmHg	None listed	Poor compliance with PD and medication	AED, intubation, BP control, and increased PD dose
**Girisgen *et al *,**[15]	11 yo male	HA, agitation, seizure	135/95 mmHg 160/100 mmHg	Erythropoietin	Underlying infection	BP control, volume control, and treatment of infection
**Delanty *et al *,**[25]	12 yo male	HA, lethargy, visual disturbances, seizure	220/120 mmHg	Erythropoietin	None given	AED and discontinuation of Erythropoietin

## Conclusion

This is to the best of our knowledge the third report of PRES in an adult patient receiving PD. The cause of this patient’s episode of PRES was likely multifactorial, stemming from the complications of ESRD, poor compliance with PD, medication side effects and possibly withdrawal. We reviewed the literature surrounding PRES and PD and found that the presentation, treatment, and final resolution of this case is consistent with the PRES literature. We feel that it is highly likely that all ESRD patients, including those receiving PD, are at an increased risk of developing PRES due to ongoing hypertension, endothelial dysfunction, and medication complications. It is also plausible that PRES is under recognized in ESRD patients, and only the extreme cases are brought to medical attention, or alternatively, are recognized by healthcare providers. It is therefore important for physicians caring for ESRD patients to consider PRES in all patients presenting with neurologic complaints. PRES is highly treatable when recognized early, and as the name suggests, is reversible; however, longstanding neurologic sequelae can be had if it is not recognized and addressed early on.

## Consent

Written informed consent was obtained from the patient for publication of this Case report and any accompanying images. A copy of the written consent is available for review by the Editor of this journal.

## Competing interests

The authors declare that they have no competing interests.

## Authors’ contributions

BG and GP were both involved in the care of the patient in this report. BG prepared the manuscript and GP proofread and revised it. Both authors read and approved the final manuscript.

## Authors’ information

BG is a neurology resident at the University of Saskatchewan. GP is a clinical professor in the Department of Medicine, Division of Nephrology, at the University of Saskatchewan.

## Pre-publication history

The pre-publication history for this paper can be accessed here:

http://www.biomedcentral.com/1471-2369/15/10/prepub
